# Regulatory Mechanisms of *ArAux*/*IAA13* and *ArAux*/*IAA16* in the Rooting Process of *Acer rubrum*

**DOI:** 10.3390/genes14061206

**Published:** 2023-05-31

**Authors:** Huiyu Zhu, Huiju Li, Jiayu Yu, Hewen Zhao, Kezhong Zhang, Wei Ge

**Affiliations:** 1 Beijing Advanced Innovation Center for Tree Breeding by Molecular Design, Beijing University of Agriculture, Beijing 102206, China; 2 College of Landscape Architecture, Beijing University of Agriculture, Beijing 102206, China; 3 Beijing Laboratory of Urban and Rural Ecological Environment, Beijing 102206, China

**Keywords:** *A. rubrum*, adventitious roots, Aux/IAA, ARF, molecular mechanisms

## Abstract

*Acer rubrum* is difficult to root during cutting propagation. Auxin/indole-acetic acids (Aux/IAA) proteins, which are encoded by the early response genes of auxin, are transcriptional repressors that play important roles in auxin-mediated root growth and development. In this study, *ArAux*/*IAA13* and *ArAux*/*IAA16*, which were significantly differentially expressed after 300 mg/L indole butyric acid treatment, were cloned. Heatmap analysis revealed that they might be associated with the process of adventitious root (AR) growth and development mediated by auxin. Subcellular localization analysis showed that they performed their function in the nucleus. Bimolecular fluorescence complementation assays revealed the interactions between them and two auxin response factor (ARF) proteins, ArARF10 and ArARF18, confirming their relevance to AR growth and development. Overexpression of transgenic plants confirmed that the overexpression of *ArAux*/*IAA13* and *ArAux*/*IAA16* inhibited AR development. These results help elucidate the mechanisms of auxin-mediated AR growth and development during the propagation of *A. rubrum* and provide a molecular basis for the rooting of cuttings.

## 1. Introduction

*A. rubrum* belongs to the Sapindaceae family and is also known as red maple, scarlet maple, and swamp maple. It is native to the northeastern United States and is mainly distributed in the northern part of the United States and most of Canada. It is a landscape tree that is used for pollution control. It is also an important street tree [1,2] (Guo et al., 2021; Zhang, 2022). *A. rubrum* is extremely difficult to root under natural conditions. There are two main methods for the asexual propagation of red maple, namely cutting and grafting. At present, the survival rate of grafted red maple is higher than that of cuttings, though the survival rate of grafting is only 80% [3] (Li, 2018). As the grafting propagation process is tedious, time-consuming, and labor-intensive, it is necessary to select and purchase high-affinity rootstocks with scions. Later management also requires a lot of labor and material resources [4] (Li et al., 2019), and so compared with grafting propagation, cutting propagation is more effective in propagating *A. rubrum* in China at present.

Currently, the propagation techniques of cuttings both in China and abroad are relatively advanced and their principles have been analyzed relatively thoroughly. Plant hormones play an important role in the rooting of plant cuttings. The main plant hormone is auxin, which promotes the growth of plant roots and is one of the most important plant hormones. Adventitious roots (ARs) are mainly dependent on the induction of exogenous hormones, mainly 3-indoleacetic acid (IAA), indole butyric acid (IBA), and naphthalene acetic acid (NAA), which regulate the occurrence of the ARs mainly by influencing the distribution of plant hormones within the spike [5] (Ma et al., 2020). One study found that the application of exogenous auxin significantly increased the rooting rate of *A. rubrum*, and IAA was particularly closely related to plant rooting [6,7,8] (Zhang et al., 2017; Hao et al., 2009; Yuan et al., 2012). A large number of studies have shown that auxin is involved in most growth and developmental reactions in plants, and plants achieve complex functions mainly through auxin anabolism, polar transport, and signal transduction, in which the most typical signal transduction regulatory pathway is that auxin regulates the growth of plant root organs [9] (Ma et al., 2020). Dynamic changes in the auxin levels of this pathway can precisely regulate the expression of downstream genes of the pathway in which it is located, such as the Gretchen Hagen 3 (GH3), Auxin/Indole-Acetic Acids protein (Aux/IAA) gene family, Auxin Response Factor (ARF) gene family, and Small Auxin-Up RNA (SAUR) family [10] (Maitra Majee et al., 2020). The three families, Aux/IAA, GH3, and SAUR, are the families of genes that regulate the early auxin response [11] (Liu et al., 2019). Briefly, in this pathway, auxin acts as a binder, binding members of the Aux/IAA protein family to F-box proteins in the Transport Inhibitor Resistant 1/Auxin signaling F-Box (TIR1/AFB) protein family, which also interacts with Skp1, while Skp1 binds to Cullin-RBX1 dimers [12] (Leyser, 2018). The F-box is part of the SCF ubiquitin ligase complex, which releases activated ubiquitin (Ub) from the E1/E2 enzyme system and transfers it to the Aux/IAA proteins to ubiquitinate them, ultimately causing the Aux/IAA proteins to be degraded [13] (Zhu et al., 2021). When the concentration of auxin in plants is low, Aux/IAA proteins bind to the ARF transcription factors and together with the co-repressor TOPLESS (TPL) proteins repress the activity of the transcription factor ARFs, thus inhibiting the transcription of the auxin response genes. When the concentration of auxin is high, Aux/IAA proteins bind to the TIR1/AFB complex, which is eventually ubiquitinated and degraded by 26S protease, and eventually ARFs are released and bind to the AuxREs binding sites of the downstream auxin response genes, regulating the transcription of the auxin response genes [14] (Quintana-Escobar et al., 2019).

Interestingly, the proteins encoded by the Aux/IAA gene family, which is an important protein family in key plant hormone regulatory pathways, have now been identified as short-lived nuclear proteins that inhibit the activity of the ARF protein family [15] (Luo et al., 2018). Aux/IAA proteins usually contain four conserved domains. Domain I is a repressor domain containing a repeated motif of leucine (LxLxL) that recognizes and binds to the co-repressor TPL to co-repress the activity of ARF proteins [16] (Liu et al., 2017). Domain II is a highly conserved domain with a “GWPPV” motif that can be recognized and bound by the TIR1/AFB complex and eventually ubiquitinates the Aux/IAA proteins and plays an important role in protein stability [17] (Paul et al., 2016). Domain III and domain IV are involved in the homo- and hetero-dimerization of Aux/IAAs with ARFs [11] (Liu et al., 2019). The Aux/IAA gene family has been identified in several species, such as *Glycine max* [18] (Ali et al., 2022), *Carica papaya* [16] (Liu et al., 2017), *Arabidopsis thaliana* [14] (Quintana-Escobar et al., 2019), and *Prunus persica* [19] (Guan et al., 2019). A total of 65 members of the Aux/IAA gene family were recently identified in *Raphanus sativus*, of which six members of this family have high expression in the cortex, formative layer, and root tip of radish roots, presumably associated with root thickening. *RsIAA33* in particular inhibits reproductive growth and promotes the thickening of the main radish roots [20] (Xie et al., 2021). In *Oryza sativa*, it has been shown that root growth and development are mainly regulated by the interaction between OsIAA13 and OsARF19 [21] (Yamauchi et al., 2019). It has even been hypothesized that AtIAA14 in *A. thaliana* interacts with the AtDi19-3 protein to promote lateral root formation, while the *AtIAA14* mutant *slr1* has a significant inhibitory effect on root growth induced by cold stress [10,22] (Maitra Majee et al., 2020; Aslam et al., 2020). However, we have not studied the key regulatory pathways of auxin in *A. rubrum*, and few reports have studied the Aux/IAA gene family in *A. rubrum*. Furthermore, there is no report on the regulatory mechanism of the interaction between Aux/IAAs and ARFs on the rooting of *A. rubrum*.

To improve the survival rate of *A. rubrum* cuttings, the rooting mechanism of maple from the molecular level of key regulatory pathways of auxin will be discussed herein. In our previous studies, 17 members of the Aux/IAA gene family were identified from the *A. rubrum* ‘Autumn Fantasy’ transcriptome by treating annual spikes with IBA and taking cuttings [13] (Zhu et al., 2021). We selected two genes, *ArAux*/*IAA13* and *ArAux*/*IAA16*, for which gene expression was significantly down-regulated, and we hypothesized that they played a significant role in the rooting of maple cuttings. Therefore, in this paper, we performed transgenic functional verification and biochemical analysis of *ArAux*/*IAA13* and *ArAux*/*IAA16* to investigate the roles of the two genes in the rooting mechanism of *A. rubrum*. Because ArARF10 [23] (Zhu et al., 2022) and ArARF18 have been successfully demonstrated to participate in plant hormone signaling pathways and promote the growth of ARs in plants, they were selected as the ArAux/IAA13 and ArAux/IAA16 interacting proteins for related experiments in this study. This study will provide further insight into the mechanism of ARs generation of *A. rubrum* ‘Autumn Fantasy’, which will be important for the propagation of *A. rubrum* mass cuttings in the future.

## 2. Materials and Methods

### 2.1. Plant Materials

The cuttings selected for this study were obtained from *A. rubrum* ‘Autumn Fantasy’ from the nursery of Landscape Architecture, Beijing University of Agriculture. We selected annual young branches as the test materials. The annual branches were cut into spikes of about 8–10 cm in length, and the upper ports of these spikes were cut flat, and the lower ports were cut diagonally, keeping half of the leaves at the tip. They were then placed into a solution of KMnO_4_ at a concentration of 0.5% for sterilization. Afterward, based on a previous study by our research group, these spikes were divided equally into two groups, one of which was inserted into water and the other into a solution containing IBA at a concentration of 300 mg/L, soaked for 1 h, and then inserted into a pre-sterilized substrate (grass carbon: vermiculite = 3:1). They were, respectively, named CK and IBA300. The room temperature was controlled at 18–25 °C, the air humidity was maintained at 60%, and the soil humidity was 80%. Ten days later, the phloem within 3 cm of the base of the spike was extracted. The removed phloem of the *A. rubrum* branches was wrapped in tin foil and stored at −80 °C in the refrigerator for subsequent experiments.

### 2.2. Bioinformatics Analysis of ArAux/IAA13 and ArAux/IAA16

The gene expression data of the ArAux/IAA gene family members were selected from the transcriptome data [23] (Zhu et al., 2022) analyzed earlier by our research group, and the data of CK and IBA300 were summed and averaged to construct a gene expression heatmap using TBtools (v1.108) software.

### 2.3. Gene Cloning

The *A. rubrum* phloem samples stored at −80 °C were subjected to total RNA extraction with The Polysaccharide Polyphenol RNA Rapid Extraction Kit (Abelson, Beijing, China). First, a clean blade was used to gently scrape off the phloem of the frozen *A. rubrum* branches, following which the total RNA in the phloem of the branches was extracted using the liquid nitrogen grinding method, and then electrophoresis was used to evaluate the extraction quality of the total RNA. Total RNA of good quality was extracted for cDNA first-strand synthesis using TransScript One-Step gDNA Removal and cDNA Synthesis SuperMix (TransGen, Beijing, China).

Primers for cloning were designed for *ArAux*/*IAA13* and *ArAux*/*IAA16* using Primer3Plus (https://www.primer3plus.com URL (accessed on 23 March 2021)) online software. Subsequently, gene cloning was performed through polymerase chain reaction (PCR) expansion.

### 2.4. Subcellular Localization

The online prediction software ProtComp 9.0 (http://linux1.softberry.com/berry.phtml URL (accessed on 21 October 2021)) was used for predicting the subcellular localization of ArAux/IAA13 and ArAuxIAA16.

The cloned *ArAux*/*IAA13* and *ArAux*/*IAA16* genes with full-length coding sequences (CDSs) were constructed into *ArAux*/*IAA13*-GFP and *ArAux*/*IAA16*-GFP recombinant vectors through Nimble Cloning (NC) reaction with the pNC-Cam1304-SubN [24] (Yan et al., 2020) vector (with GFP tag sequence). The successfully constructed *ArAux*/*IAA13*-GFP and *ArAux*/*IAA16*-GFP recombinant vectors were transformed into *Agrobacterium rhizogenes* GV3101. A single colony was incubated in YEB liquid medium (containing Rif and Kan antibiotics) until the optical density at 600 nm (OD600) was 0.6, and then the bacterial solution was configured as an infestation solution, then, it was co-transfected into tobacco leaf cells with nucleus marker. Low light culture for two days after infection. Subsequently, the protoplasts are extracted. Then, the green fluorescent protein (GFP) signal was observed with a fluorescent orthomosaic microscope (Nikon C2-ER, Shanghai, China) at an excitation wavelength of 488–580 nm. A negative control was also set up.

### 2.5. Bimolecular Fluorescence Complementation (BIFC) Assay

For bimolecular fluorescence complementation detection, the complete CDSs of the *ArARF10* and *ArARF18* genes were inserted into the pNC-BIFC-ENN [24] (Yan et al., 2020) vector (with NYFP tag sequence) to constitute the *ArARF10*-N-YFP recombinant vector and *ArARF18*-N-YFP recombinant vector. The complete CDSs of the *ArAux*/*IAA13* and *ArAux*/*IAA16* genes were inserted into the pNC-BIFC-ECN [24] (Yan et al., 2020) vector (with CYFP tag sequence) to constitute the *ArAux*/*IAA13*-C-YFP recombinant vector and *ArAux*/*IAA16*-C-YFP recombinant vector. The four constructed recombinant vectors were transformed into *A. rhizogenes* GV3101. The transformed *Agrobacterium* was cultured in liquid medium YEB (containing Rif and Kan antibiotics) until the OD600 was 1.0 and then configured into an infestation solution. The infestation solution was mixed 1:1 according to the test combination (Table 1) and dark-treated for 3 h before being injected into the leaves of Ben’s tobacco at 5–6 weeks of breeding age. After dark treatment for 24 h and incubation for three days, the yellow fluorescent protein (YFP) signal was observed in the lower epidermis of the tobacco leaves using a laser confocal microscope (Leica TCS SP5, Germany) (excitation wavelength of 514 nm). The test combinations with empty carriers were used as negative controls.

### 2.6. Transgenic Functional Validation

The T-DNA insertion patterns of the *A. thaliana* loss-of-function mutants *aux*/*iaa13* and *aux*/*iaa16* were purchased from the Chinese *A. thaliana* Mutant Sharing Center (AraShare, Fuzhou, China). The full CDSs length of the *ArAux*/*IAA13* and *ArAux*/*IAA16* genes was inserted into the pNC-Cam3304-MCS35S [24] (Yan et al., 2020) overexpression vector by NC reaction to constitute *ArAux*/*IAA13*-pCamiba3304 and *ArAux*/*IAA16*-pCamiba3304 recombinant vectors to obtain overexpressed *ArAux*/*IAA13* and *ArAux*/*IAA16* plants (OE-*ArAux*/*IAA13* and OE-*ArAux*/*IAA16*). Then, we transformed the successfully constructed recombinant vector into *A. rhizogenes* GV3101. Ultimately, we used the inflorescence infestation method to infest the mutant *A. thaliana aux*/*iaa13* and *aux*/*iaa16* with the infestation solution. We identified positive seedlings when cultured to the T1 generation and collected the seeds. The successfully identified T1 generation seeds were cultured to the T2 generation, and seeds from the T2 generation were then collected. After the seeds were dried, they were stored in a refrigerator at 4 °C. A small amount of wild-type seeds, *A. thaliana* mutant seeds, and T2 generation seeds were placed in 1/2 Murashige and Skoog (MS) solid medium and vernalized at 4 °C for 3 days. After that, the seedlings were photoinduced at 19 °C for 5 h and then wrapped in three layers of tin foil in the dark at 19 °C for 4 days until the average length of the seedling hypocotyls reached 6 mm. All root organs of the seedlings were excised with a sterile blade on a sterile ultra-clean bench, leaving only the 6 –mm long hypocotyls. The treated seedlings were neatly placed in 1/2 MS solid vertical medium with clean forceps and cultivated at 23 °C for 16 h in light and 20 °C for 8 h in dark to induce ARs formation. Three biological replicates were performed for both the experimental and control groups, and the length and number of ARs were recorded. Finally, the obtained experimental results were measured for specific traits using ImageJ and statistically analyzed using GraphPad8 (paired *t*-test).

The relative expressions of *ArAux*/*IAA13* and *ArAux*/*IAA16* were verified in transgenic *A. thaliana* mutants through quantitative real-time PCR (qRT-PCR). Total RNA was extracted from the leaves of the transgenic *A. thaliana* mutant T3 generation and wild-type *A. thaliana* using the polysaccharide polyphenol RNA rapid extraction kit (Aibosun, Beijing, China). Total RNA was extracted for fluorescent quantitative cDNA first-strand synthesis using TransScript One-Step gDNA Removal and cDNA Synthesis SuperMix (TransGen, Beijing, China). Primers were designed using the online software Primer3Plus (https://www.primer3plus.com URL (accessed on 23 June 2022)) (Table 2). The enzyme used in this assay was 2× SYBR GREEN (Aiberson, Beijing, China). The qRT-PCR cycling steps were 95 °C for 2 min, followed by 95 °C for 15 s, 60 °C for 20 s, and then 72 °C for 20 s, for 45 total cycles. On average, each reaction was performed five times. The internal reference used in this assay was 18S rRNA [25] (Wang et al., 2015).

## 3. Results

### 3.1. Bioinformatics Analysis of ArAux/IAAs

**Figure 1:**
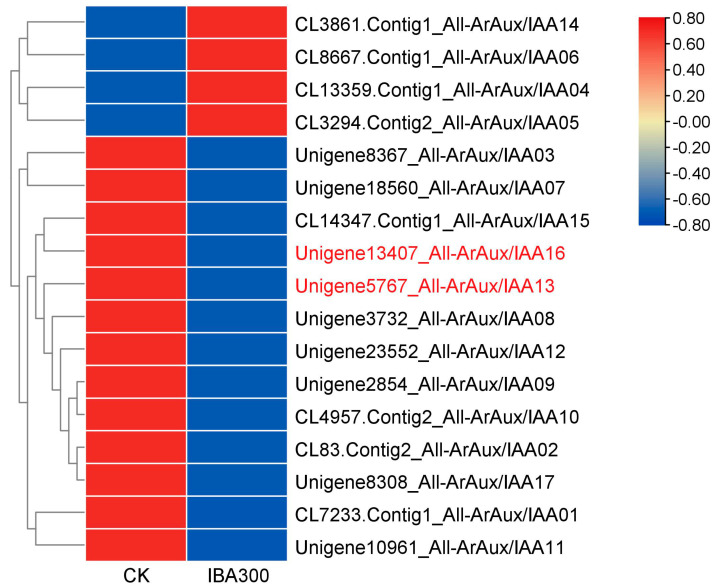
Heatmap clustering analysis of ArAux/IAAs. Bars represent scaled gene expression levels log10 (FPKM+1).

Figure 1 shows that the expression trend of ArAux/IAAs was divided into two modules: up-regulated and down-regulated trends. The three significantly up-regulated gene IDs were: *ArAux*/*IAA5* (CL3294.Contig2_All), *ArAux*/*IAA14* (CL3861.Contig2_All), and *ArAux*/*IAA6* (CL8667.Contig1_All); the three significantly down-regulated gene IDs were: *ArAux*/*IAA13* (Unigene5767_All), *ArAux*/*IAA16* (Unigene13407_All), and *ArAux*/*IAA8* (Unigene3732_All). The results showed that the expressions of red-labeled *ArAux*/*IAA13* (Unigene5767_All) and *ArAux*/*IAA16* (Unigene13407_All) were significantly down-regulated after treatment.

### 3.2. Subcellular Localization

**Figure 2:**
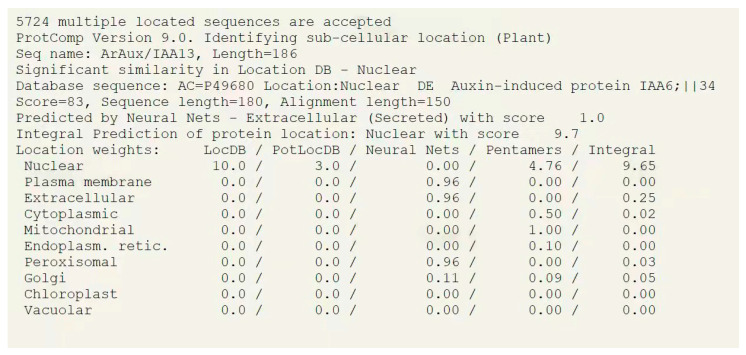
Predicted results of subcellular localization of ArAux/IAA13.

**Figure 3:**
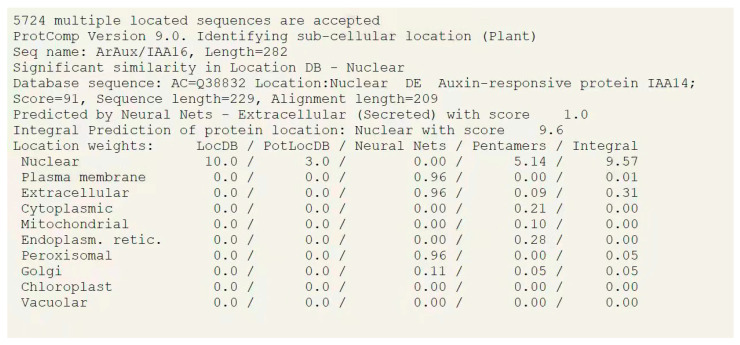
Predicted results of subcellular localization of ArAux/IAA16.

The online prediction software ProtComp 9.0 was used to predict the subcellular localization of ArAux/IAA13 and ArAuxIAA16. The results are shown in Figure 2 and Figure 3, which predict that ArAux/IAA13 and ArAux/IAA16 transcription factors may play their roles in the nucleus.

**Figure 4:**
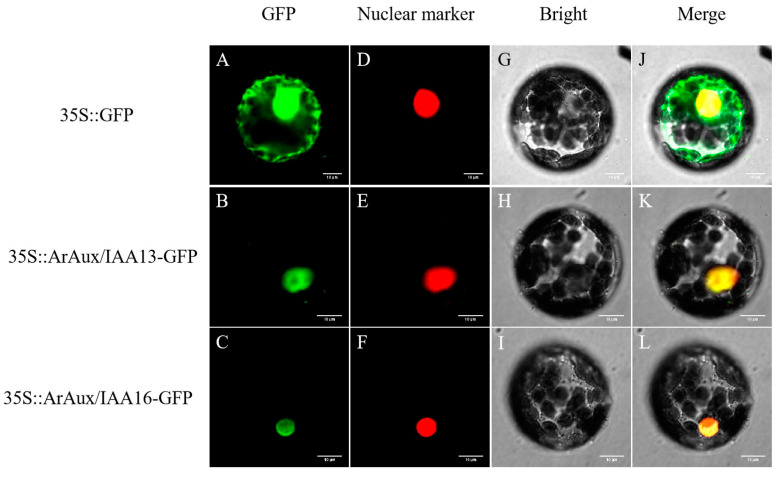
Subcellular localization of ArAux/IAA13 and ArAux/IAA16. (**A**–**C**) show the green fluorescence field, (**D**–**F**) show the nuclear marker field. (**G**–**I**) show the bright field, and (**J**–**L**) show the merged field. Scale bar, 10 μm.

The subcellular localization results indicated that the GFP green fluorescence of the *ArAux*/*IAA13*-GFP and *ArAux*/*IAA16*-GFP fusion proteins was only detected in the nucleus of the cells (Figure 4). By contrast, the GFP fluorescent protein of the control was detected in the nucleus, cell membrane, and cytoplasm. Thus, ArAux/IAA13 and ArAux/IAA16 proteins are mainly localized in the nucleus of plant cells.

### 3.3. Interaction of ArAux/IAA13 and ArAux/IAA16 with ArARF10 and ArARF18

**Figure 5:**
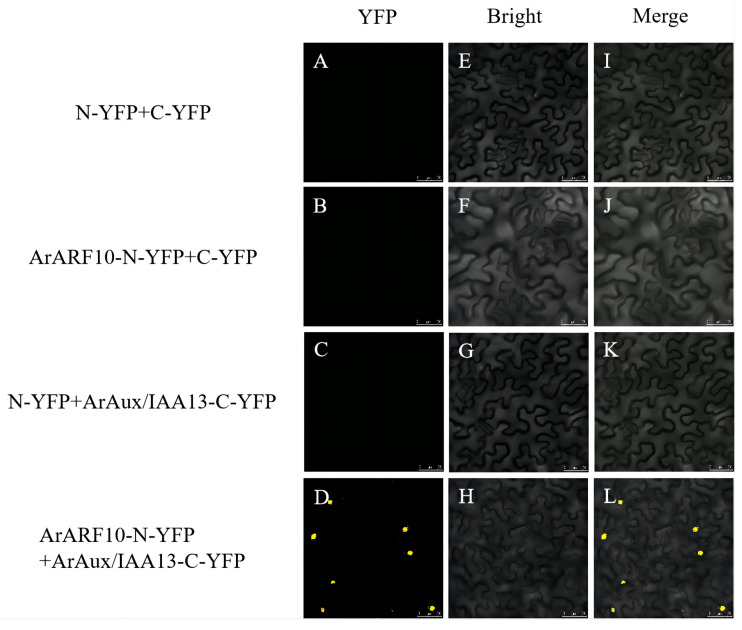
Bimolecular fluorescence complementary assay between ArARF10 and ArAux/IAA13. (**A**–**D**) show the yellow fluorescence field, (**E**–**H**) show the bright field, and (**I**–**L**) show the merged field. Scale bar, 50 μm.

**Figure 6:**
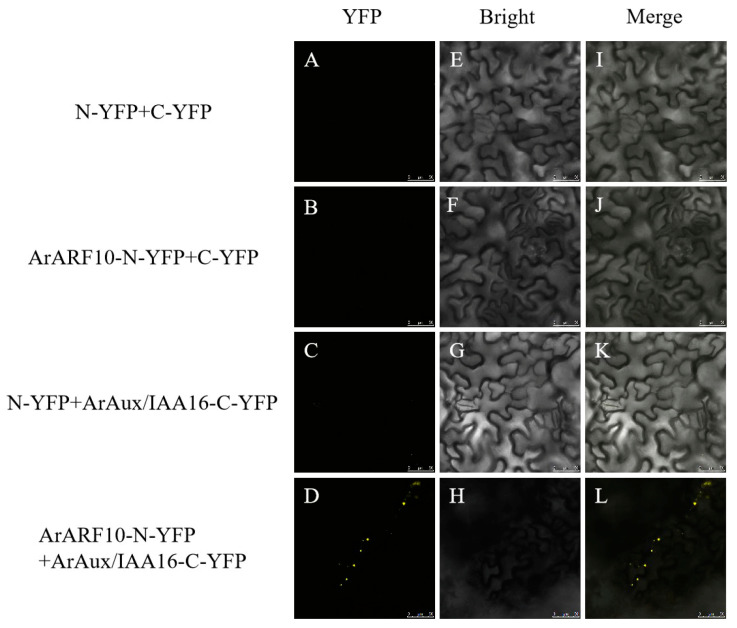
Bimolecular fluorescence complementary assay between ArARF10 and ArAux/IAA16 proteins. (**A**–**D**) show the yellow fluorescence field, (**E**–**H**) show the bright field, and (**I**–**L**) show the merged field. Scale bar, 50 μm.

**Figure 7:**
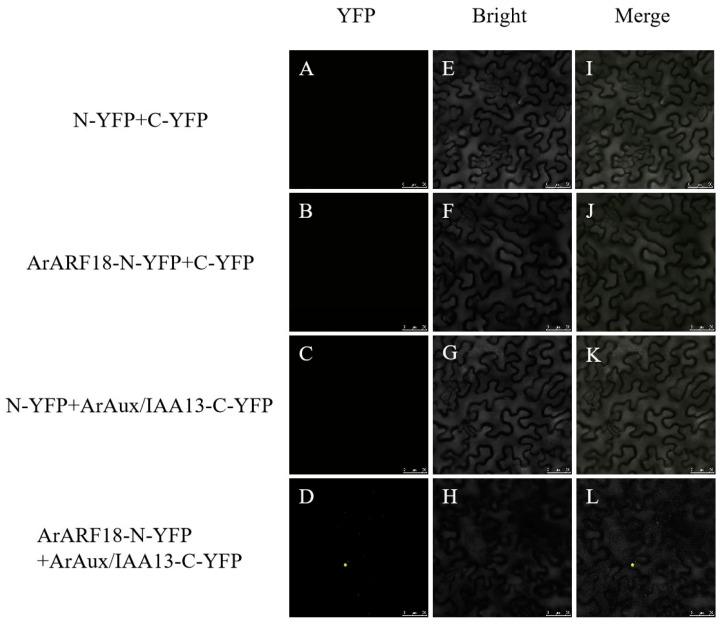
Bimolecular fluorescence complementary assay between ArARF18 and ArAux/IAA13 proteins. (**A**–**D**) show the yellow fluorescence field, (**E**–**H**) show the bright field, and (**I**–**L**) show the merged field. Scale bar, 50 μm.

**Figure 8:**
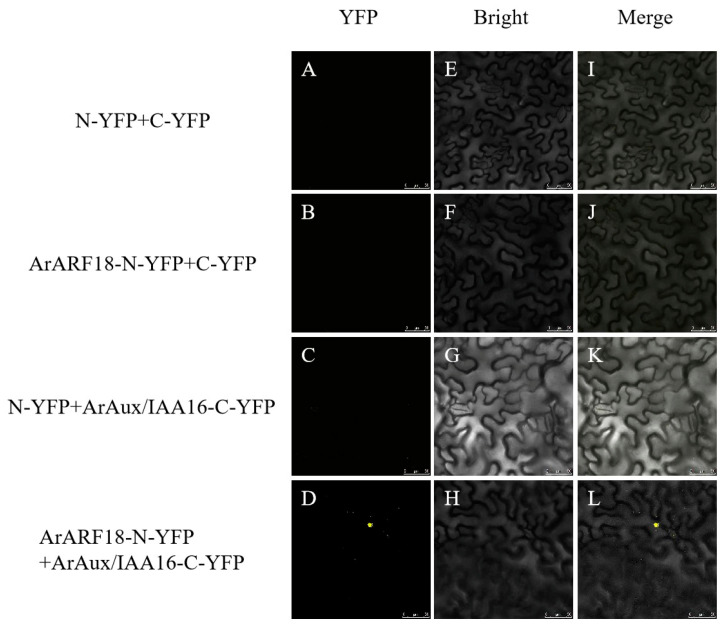
Bimolecular fluorescence complementary assay between ArARF18 and ArAux/IAA16 proteins. (**A**–**D**) show the yellow fluorescence field, (**E**–**H**) show the bright field, and (**I**–**L**) show the merged field. Scale bar, 50 μm.

Figure 5, Figure 6, Figure 7 and Figure 8 show the results of YFP expression in tobacco leaves through BIFC assay. Both ArAux/IAA13 and ArAux/IAA16 were found to interact with ArARF10, and bright yellow fluorescence was only found in the nucleus. In addition, both ArAux/IAA13 and ArAux/IAA16 were found to interact with ArARF18 in the nucleus, but the yellow fluorescence was less than that with ArARF10. This suggests that ArARF10 and ArARF18 could interact with ArAux/IAA13 and ArAux/IAA16.

### 3.4. Overexpression of ArAux/IAA13 and ArAux/IAA16 in A. thaliana Mutants

**Figure 9:**
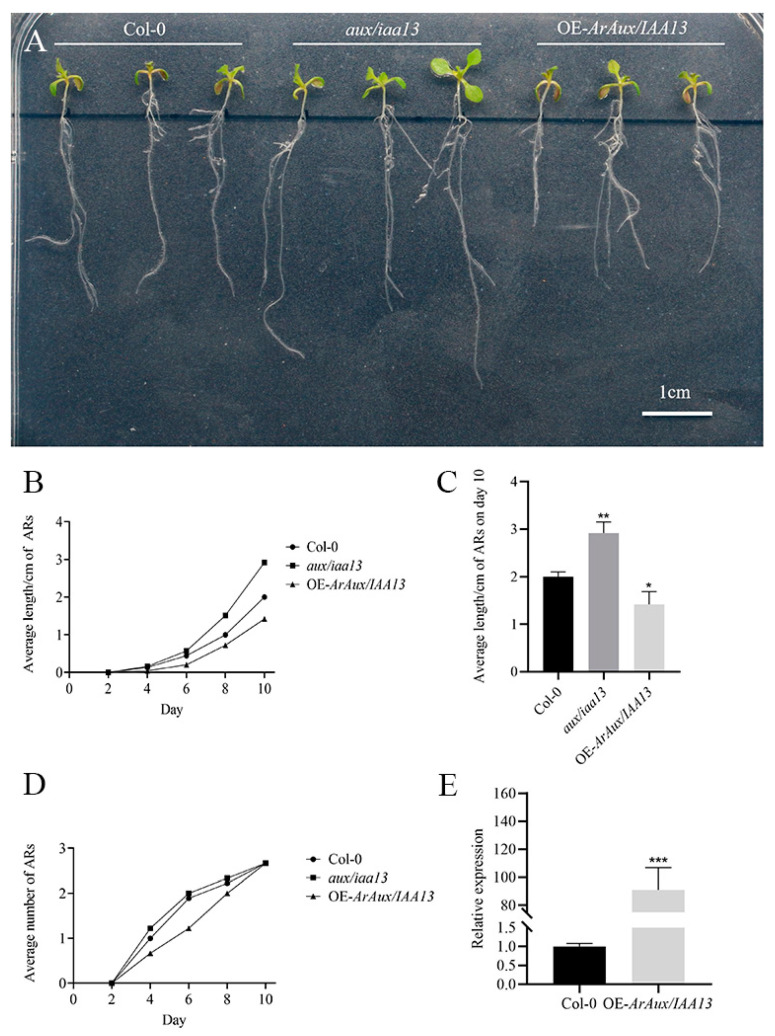
Transgenic functional validation phenotype of *ArAux*/*IAA13.* (**A**) shows the T3 phenotypic observation graph. (**B**) shows the line graph of the length/cm of ARs in 10 days. (**C**) shows the bar chart of the length/cm of ARs on the 10th day. (**D**) shows the line graph of the number of ARs in 10 days. (**E**) shows the real-time fluorescence PCR expression of *ArAux*/*IAA13* in Col-0 and OE-*ArAux*/*IAA13*. Scale bar, 1 cm. * *p* < 0.05, ** *p* < 0.01, *** *p* < 0.001, *n* = 9.

**Figure 10:**
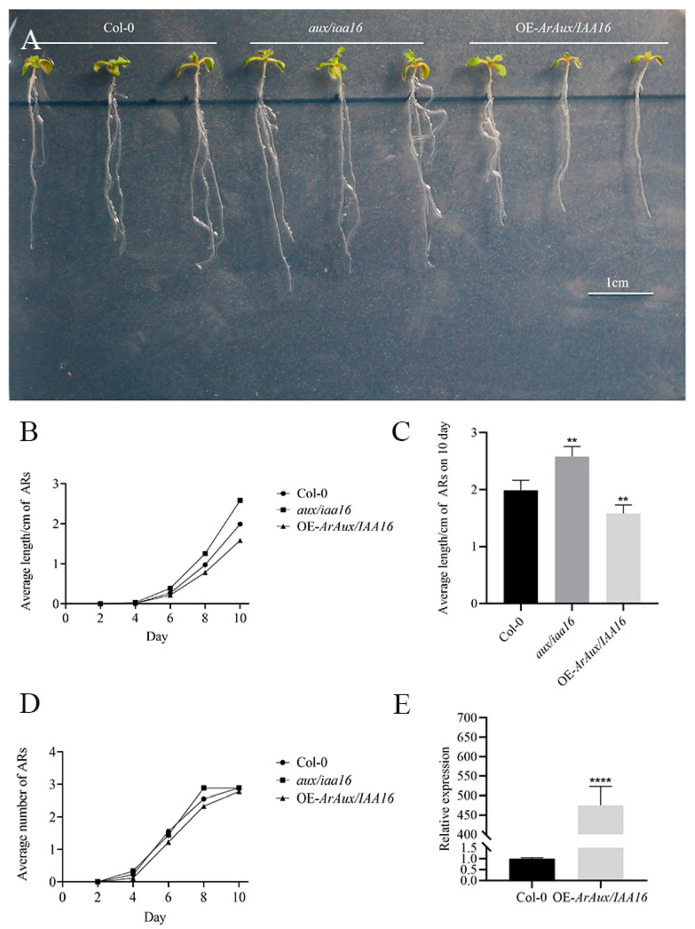
Transgenic functional validation phenotype of *ArAux*/*IAA16*. (**A**) shows the T3 phenotypic observation graph. (**B**) shows the line graph of the length/cm of ARs in 10 days. (**C**) shows the bar chart of the length/cm of ARs on the 10th day. (**D**) shows the line graph of the number of ARs in 10 days. (**E**) shows the real-time fluorescence PCR expression of *ArAux*/*IAA16* in Col-0 and OE-*ArAux*/*IAA16*. Scale bar, 1 cm. ** *p* < 0.01, **** *p* < 0.0001, *n* = 9.

To verify the role of *ArAux*/*IAA13* and *ArAux*/*IAA16* in the growth and development of ARs in plants, transgenic transfer was performed in A. thaliana mutants and root growth was then observed. Through transgenic functional verification, the ARs of Col-0, *aux*/*iaa13*, and OE-*ArAux*/*IAA13* (Figure 9A), and Col-0, *aux*/*iaa16*, and OE-*ArAux*/*IAA16* (Figure 10A) were examined after 10 days of cultivation. The results showed that there were obvious phenotypic differences in the length of the ARs of Col-0 and *aux*/*iaa13* and *aux*/*iaa16*. The length of the ARs of *aux*/*iaa13* and *aux*/*iaa16* was significantly longer than that of Col-0 (Figure 9C and Figure 10C). The ARs of OE-*ArAux*/*IAA13* and OE-*ArAux*/*IAA16* were significantly shorter than *aux*/*iaa13* and *aux*/*iaa16* and were also shorter than Col-0 (Figure 9C and Figure 10C). The AR number and length of the mutants of both genes were overall higher than the Col-0 and overexpression plants during these 10 days, while those of the overexpression plants were overall lower than the mutants and Col-0. On day 6, the number of ARs of OE-*ArAux*/*IAA13* differed the most from that of Col-0 and *aux*/*iaa13* (Figure 9D).

To verify overexpression, qRT-PCR was performed. The qRT-PCR results indicated that the relative expression levels of *ArAux*/*IAA13* and *ArAux*/*IAA16* in the T3 transgenic plants were significantly higher than those of Col-0 (Figure 9E and Figure 10E). The expression level of OE-*ArAux*/*IAA13* was about 90 times higher than that of Col-0 and this difference was significant. The expression level of OE-*ArAux*/*IAA16* was about 450 times higher than that of Col-0 and this difference was also significant. Thus, the two genes were transferred into the *A. thaliana* mutant and successfully overexpressed.

## 4. Discussion

This paper studied the role of *ArAux*/*IAA13* and *ArAux*/*IAA16* in the key regulatory pathway of plant hormone signaling in *A. rubrum* and in the regulation of maple rooting. Over the past few years, the Aux/IAA family has been identified in many species, such as *Eucalyptus robusta* [26] (Yu et al., 2015), *Cucumis sativus* [27] (Gan et al., 2013), *Zea mays* [28] (Wang et al., 2010), *G. max* [29] (Singh et al., 2015), and *P. persica* [19] (Guan et al., 2019), indicating that this family plays a critical role in the plant hormone signaling pathway.

In this study, the clustering heatmap analysis of the ArAux/IAAs following IBA 300 mg/L treatment showed that the gene expression of the ArAux/IAAs of *A. rubrum* was divided into two modules of up-regulation and down-regulation, indicating that the ArAux/IAAs had multiple modes of regulation. This family exhibits similar gene expression patterns in most species. A total of 44 genes of the Aux/IAA family have been identified in *Arachis hypogaea*, of which 31 genes of this family were differentially expressed in *A. hypogaea* seeds, with the up-regulated expression of *AhIAA-3A*, *AhIAA-16A*, and *AhIAA-15B* and down-regulated expression of *AhIAA-11A*, *AhIAA-5B*, and *AhIAA-14B* [30] (Zhang et al., 2022). In *Solanum lycopersicum*, six genes (*SlIAA11*, *SlIAA15*, *SlIAA16*, *SlIAA17*, *SlIAA19*, and *SlIAA23*) were up-regulated, while three genes (*SlIAA20*, *SlIAA21*, and *SlIAA22*) were significantly down-regulated after exogenous auxin treatment [31] (Wu et al., 2012). This experiment also revealed that Aux/IAAs are sensitive to the auxin response factors and the key genes in plant root development [32] (Hochholdinger et al., 2018). In this paper, the expression of *ArAux*/*IAA13* and *ArAux*/*IAA16* showed significant down-regulation patterns and combined with the abovementioned research results, we speculated that they played negative regulatory roles in the rooting of *A. rubrum*. The proteins encoded by the Aux/IAA gene family are generally identified as negative regulators during rooting in most plants and act as inhibitors of rooting, which is consistent with our research. In *A. thaliana*, Aux/IAAs inhibited hypocotyl growth through photoreceptors [33] (Li et al., 2022), and three proteins, namely AtIAA6, AtIAA9, and AtIAA17, were found to have cumulative effects in inhibiting AR development [34] (Lakehal et al., 2019).

Most transcription factors are located in the nucleus and perform their functions there. Several studies have shown that Aux/IAA proteins are short-lived nuclear proteins, with ZmIAA2, ZmIAA11, and ZmIAA15 in *Z. mays* [35] (Ludwig et al., 2014) and PeIAA8 in *Phyllostachys edulis* [36] (Li et al., 2018) all localized in the nucleus. This is because Aux/IAA proteins that localize in the nucleus all contain nuclear localization signals that direct the proteins into the nucleus to perform their functions [37,38] (Abel et al., 1994; Wu et al., 2017). In this study, ArAux/IAA13 and ArAux/IAA16 were also confirmed to be localized in the nucleus to perform their functions through the subcellular localization assay.

Aux/IAAs and ARFs are two key transcription factors in the auxin regulatory pathway, and the auxin signaling model predicted that auxin was largely dependent on ARF-Aux/IAA interactions. The interaction between the two proteins is mediated by the protein–protein interaction domain (CTD) at the C-terminus of ARF and Aux/IAA proteins. In this study, we successfully verified that ArAux/IAA13 and ArAux/IAA16 interact with ArARF10 and ArARF18 through the BIFC assay, in which ArAux/IAA13 and ArAux/IAA16 significantly interacted with ArARF10. By analyzing the ArAux/IAA13 and ArAux/IAA16 protein modules, we found that they both contained motif 1 and motif 2 in two conserved domains: domain III and domain IV. Recent studies have indicated the existence of a high degree of compensation among Aux/IAA genes. In *A. thaliana*, one study [39] (Ori, 2019) produced loss-of-function double and triple mutants, which were not significantly different compared with Col-0. However, in *S. lycopersicum* [40] (Wang et al., 2005), missing an Aux/IAA family gene resulted in a distinct phenotype; however, dominant mutants in this gene family would have a more pronounced phenotype. This is similar to the case of the *A. thaliana* loss-of-function mutants used in this study that did not differ significantly from the wild type in AR occurrence. This phenomenon may be related to the species and the mode of mutation. The acquired mutant of the *AtIAA16* gene in *A. thaliana* has now been shown to have an inhibitory effect on plant growth, while it can make plants less sensitive to auxin [41] (Rinaldi et al., 2012). In *Vaccinium* spp. [42] (Hou et al., 2020), it has been found that the overexpression of *VcIAA27* in *A. thaliana* leads to leaf curling and plant dwarfism, while it is speculated to play a negative regulatory role in the auxin regulatory pathway. In *C. papaya* [43] (Estrella-Maldonado et al., 2022), the rooting rate increased significantly after exogenous hormone treatment, in which CpAux/IAA family expression was down-regulated, while CpARF family expression was up-regulated, indicating that the Aux/IAA gene family also had a negative regulatory role in the auxin signaling pathway in *C. papaya*. This is similar to the situation whereby the length of the ARs of the overexpression OE-*ArAux*/*IAA13* and OE-*ArAux*/*IAA16* in this paper was lower than that of the wild-type and mutant *A. thaliana*. Furthermore, the lengths of the ARs of the deletion-type mutants were much higher than those of the Col-0 and transgenic *A. thaliana*. In conclusion, this confirmed that the Aux/IAA family plays a negative role in auxin-regulated plant rooting.

Thus far, many studies have confirmed that the ARF family promotes root growth and development. In addition, our research group has successfully proved that *ArARF10* [23] (Zhu et al., 2022) and *ArARF18* in *A. rubrum* can promote the growth and development of plant ARs. In this study, we found that ArAux/IAA13 and ArAux/IAA16 can interact with ArARF10 and ArARF18, and we successfully verified that ArAux/IAA13 and ArAux/IAA16 have an inhibitory effect on plant AR growth. This indicates that ArAux/IAA13 and ArAux/IAA16 mainly inhibit the downstream gene transcription of ArARFs through interaction with ArARFs in the nucleus. This also indicates that they are involved in the regulatory role of key regulatory pathways of plant hormone signal transduction related to root growth and development. They regulate the growth and development of plant ARs by interacting with ArARFs.

## 5. Conclusions

*ArAux*/*IAA13* and *ArAux*/*IAA16* play an important role in the growth and development of ARs in *A. rubrum*. Heatmap analysis indicated that they were significantly down-regulated after 300 mg/mL IBA treatment. Subcellular localization confirmed that ArAux/IAA13 and ArAux/IAA16 were located in the nucleus. The bimolecular fluorescence complementary test showed that they could interact with ArARF10 and ArARF18, which further verified their regulatory mechanisms in the auxin regulation pathway. Furthermore, the functional verification of transgenes also confirmed that they played negative regulatory roles in AR growth and development. This research provides new avenues and data for the study of the rooting regulation of *Acer*.

## Figures and Tables

**Table 1:** Bimolecular fluorescence combination of ArARF10, ArARF18, ArAux/IAA13, and ArAux/IAA16. 1–5 are the negative controls, and 6–9 are the test groups.

Serial Number	ENN-Linked Genes	ECN-Linked Genes
1	None	*ArAux*/*IAA13*
2	None	*ArAux*/*IAA16*
3	*ArARF10*	None
4	*ArARF18*	None
5	None	None
6	*ArARF10*	*ArAux*/*IAA13*
7	*ArARF18*	*ArAux*/*IAA13*
8	*ArARF10*	*ArAux*/*IAA16*
9	*ArARF18*	*ArAux*/*IAA16*

**Table 2:** Primers used in qRT-PCR.

Primer Names	Primer Sequences
18S-F	CCTGAGAAACGGCTACCACAT
18S-R	CACCAGACTTGCCCTCCA
*ArAux*/*IAA13*-F	GAAAGTCCAGACGAATGAGAGC
*ArAux*/*IAA13*-R	TCCAACGTCATGGCAAGATC
*ArAux*/*IAA16*-F	AACAAGAAGAAAGGAGGCACAG
*ArAux*/*IAA16*-R	CCAGAGAAAACCCACTTGCTAT

## Data Availability

All relevant data are contained within the manuscript text, and any additional underlying data are available upon request.
